# Dietary Intake and Chronic Disease Prevention

**DOI:** 10.3390/nu13041358

**Published:** 2021-04-19

**Authors:** Annalisa Noce, Annalisa Romani, Roberta Bernini

**Affiliations:** 1 UOC of Internal Medicine-Center of Hypertension and Nephrology Unit, Department of Systems Medicine, University of Rome Tor Vergata, Via Montpellier 1, 00 133 Rome, Italy; 2 PHYTOLAB (Pharmaceutical, Cosmetic, Food Supplement, Technology and Analysis), DiSIA, University of Florence Via Ugo Schiff 6, 50 019 Florence, Italy; annalisa.romani@unifi.it; 3 Department of Agriculture and Forest Sciences (DAFNE), University of Tuscia, Via San Camillo de Lellis, 01 100 Viterbo, Italy; roberta.bernini@unitus.it

Non-communicable diseases (NCDs) are non-infectious chronic pathologies. The most common are diabetes mellitus, obesity, metabolic syndrome, chronic kidney disease (CKD), cardiovascular (CV) diseases, cancer, and chronic respiratory diseases. Furthermore, their prevalence is likely to increase over time due to the aging population, urbanization, and lifestyle changes [1]. In industrialized and high-income countries, several studies have highlighted a direct correlation between socio-economic factors and health status; in particular, NCDs affect mainly the population with the lowest socio-economic level [2,3,4,5]. 

Before the COVID-19 pandemic, NCDs had spread all over the world, becoming an important public health problem even in developing countries. The “epidemiologic transition” observed from infectious diseases to NCDs in developing countries is related to a series of risk factors, mainly associated with economic development, such as the consumption of foods with high contents of saturated fats, salt, and sugars; low intake of fruit, vegetables, fiber, and ω-3 fatty acids; a sedentary lifestyle; smoking; and the unmoderated consumption of alcohol [6,7]. 

NCDs are responsible for high percentages of disability and mortality worldwide [8]. 

An unhealthy lifestyle, characterized by an unbalanced diet, together with insufficient sleep, physical inactivity, psychological stress, environmental pollution [9], smoking, or alcohol abuse contribute to cause metabolic alterations which can lead to the onset of NCDs. 

In this context, a correct lifestyle and healthy dietary habits could exert protective effects, increasing the life expectancy. Then, nutrition plays an important role in NCDs prevention [10]. In particular, the Mediterranean diet, characterized by a high consumption of fruit, vegetables, extra virgin olive oil, cereals, legumes, and fish; a moderate intake of dairy products, eggs, and red wine; and a low intake of animal fats and red meat, represents a correct approach to prevent NCDs onset [11,12,13,14,15,16]. Moreover, pasta represents one of the basic foods of Mediterranean diet and, in this Special Issue, a preliminary study analyzes the antioxidant compounds present in three types of pasta and their biological activities on kidney cells, demonstrating that pasta’s natural bioactive compounds play positive role in the protection of kidney cells from oxidative stress [17].

The beneficial effects are related to the presence of natural bioactive compounds, including antioxidants [18]. Epidemiological studies have demonstrated that an optimal daily intake of antioxidants such as polyphenols and vitamins is able to counteract the onset of NCDs and to slow their progression [18]. Polyphenols are a wide and complex group of compounds found in plant-derived foods, beverages, and agro-industrial byproducts; these bioactive molecules have important physiological effects on the prevention of several chronic diseases. For example, small phenols such as hydroxytyrosol found in extra-virgin olive oil and olive oil byproducts, catechins such as epigallocatechin found in green tea, and complex hydrolysable tannins such as punicalagin found in pomegranate peel and fruit, exhibit strong antioxidant, anti-inflammatory, antidiabetic, anti-obesity, anticancer, and antimicrobic activities [19,20,21,22,23,24,25]. In this context, an original article of this Special Issue demonstrated the gender-dependent positive antimicrobial action of *Castanea sativa* L. hydrolysable tannins in recurrent urinary infections in CKD patients [26].

The relationship between gut dysbiosis and the onset of NCDs has recently been highlighted [27,28]. Several studies have shown that polyphenols could influence the composition of the gut microbiota by promoting the growth of bacterial classes with positive effects and by inhibiting bacteria with negative effects on the microbiota composition [29,30]. Moreover, vitamins, and, in particular, vitamin C (ascorbic acid) and E (tocopherols), are natural compounds that play a pivotal role in preventing the NCDs onset, mainly for their antioxidant activity. Vitamin C is a water-soluble vitamin, able to protect from the cellular damage exerted by harmful oxidative compounds [31]. Vitamin E includes a group of lipid-soluble compounds with the highest antioxidant activity in vivo [32].

This Special Issue has contributed to better evidence of the role of the correct lifestyle and the natural bioactive compounds in preventing the NCDs onset and their treatment. In fact, some reviews and original articles have confirmed the cardioprotective role exerted by different dietary patterns and by natural bioactive compounds [33]. In particular, how a personalized Mediterranean diet in women can exert a positive action on the cardiovascular system [34], how ω-3 polyunsaturated fatty acids play a cardioprotective role in male obesity secondary hypogonadism (MOSH) patients [35], and how a caloric restriction diet can protect against organ damage induced by arterial hypertension, improving endothelial dysfunction [36]. Another study evaluated the possible relationship between dietary quality scores and cardiometabolic risk in a group of older Australian adults, demonstrating that a high intake of vegetables, grains, and non-processed red meat was associated with a better cardiometabolic risk profile [37]. An original review stressed a relationship between frailty, sarcopenia, and cardiovascular risk, underlining how the frail phenotype is associated with a poor outcome after cardiac surgery [38]. Healthy eating habits reduce also the risk of developing cancer [39,40,41] and other chronic NCDs (such as CKD and chronic respiratory diseases) [42,43,44,45], as evidenced by several papers of this Special Issue. 

The Guest Editors would like to thank all the authors, reviewers who contributed to the success of this Special Issue, and the Nutrients team for their precious and constant support.
